# How Can the Introduction of Zr^4+^ Ions into TiO_2_ Nanomaterial Impact the DSSC Photoconversion Efficiency? A Comprehensive Theoretical and Experimental Consideration

**DOI:** 10.3390/ma14112955

**Published:** 2021-05-30

**Authors:** Aleksandra Bartkowiak, Oleksandr Korolevych, Gian Luca Chiarello, Malgorzata Makowska-Janusik, Maciej Zalas

**Affiliations:** 1 Faculty of Chemistry, Adam Mickiewicz University, Uniwersytetu Poznańskiego 8, 61-614 Poznań, Poland; 2 Department of Chemistry, University of Milan, Via Golgi 19, 20133 Milano, Italy; gianluca.chiarello@unimi.it; 3 Faculty of Science and Technology, Jan Dlugosz University, Al. Armii Krajowej 13/15, 42-200 Częstochowa, Poland; oleksandr.korolevych@doktorant.ujd.edu.pl (O.K.); m.makowska@ujd.edu.pl (M.M.-J.)

**Keywords:** DSSC, photovoltaics, TiO_2_, Zr^4+^ ions, doping, Zr^4+^ doped TiO_2_, Ti_1−x_Zr_x_O_2_

## Abstract

A series of pure and doped TiO_2_ nanomaterials with different Zr^4+^ ions content have been synthesized by the simple sol-gel method. Both types of materials (nanopowders and nanofilms scratched off of the working electrode’s surface) have been characterized in detail by XRD, TEM, and Raman techniques. Inserting dopant ions into the TiO_2_ structure has resulted in inhibition of crystal growth and prevention of phase transformation. The role of Zr^4+^ ions in this process was explained by performing computer simulations. The three structures such as pure anatase, Zr-doped TiO_2_, and tetragonal ZrO_2_ have been investigated using density functional theory extended by Hubbard correction. The computational calculations correlate well with experimental results. Formation of defects and broadening of energy bandgap in defected Zr-doped materials have been confirmed. It turned out that the oxygen vacancies with substituting Zr^4+^ ions in TiO_2_ structure have a positive influence on the performance of dye-sensitized solar cells. The overall photoconversion efficiency enhancement up to 8.63% by introducing 3.7% Zr^4+^ ions into the TiO_2_ has been confirmed by I-V curves, EIS, and IPCE measurements. Such efficiency of DSSC utilizing the working electrode made by Zr^4+^ ions substituted into TiO_2_ material lattice has been for the first time reported.

## 1. Introduction

In the last few decades, many scientific papers have focused on research on the generation and storage of energy, e.g., batteries, supercapacitors, wind turbines (wind farm), heat exchangers (geothermal energy) [1,2,3,4,5]. The year 1991 marked a significant milestone in the photovoltaic technology world due to the first highly efficient dye-sensitized solar cells (DSSCs) invented by O’Regan and Grätzel [6]. This breakthrough invention has been extensively developed for the last 30 years by worldwide scientists (about 29,770 records of scientific articles may be found in databases). Due to many advantages, such as inexpensive manufacturing costs using non-toxic substrates and leaving a remarkably lower carbon footprint, as well as workability under indoor ambient light, DSSCs are a promising alternative to the other types of solar cells [7]. It is still worth developing a technology that uses renewable energy sources such as solar light from an ecological perspective. Over 85% of humanity’s energy demand is met by fossil fuels, leading to severe climate changes such as global warming and depletion of our planet’s natural sources [8]. Moreover, the announced 2020 pandemic of coronavirus SARS-Cov-2 shows how reducing car transport (through introducing, e.g., remote learning and work) and decreasing energy consumption caused a drop in greenhouse gases emission [9]. That leads to the statement that changing some habits and a few factors can positively affect the environment. The issues mentioned above confirm the validity of supporting sustainable energy sources and developing technologies in line with the Internet of Things (IoT) concept. Unlike silicon photovoltaic cells, recent reports indicate that DSSCs can efficiently convert solar energy into electricity even under low light conditions. DSSCs work either on a cloudy day, installed on the building’s façade, or under ambient conditions, powering household appliances, which make them even more promising [10,11].

DSSCs consists of three essential parts: photoanode (usually a metalorganic dye adsorbed on a semiconductor metal oxide), electrolyte, and a counter electrode. The dye’s electron is excited by photon absorption from the ground state and injected into the semiconductor’s conduction band [12]. Subsequently, electrons from the conduction band are transported through the external circuit to the counter electrode, acting as an electron collector [13]. Finally, a redox mediator in the electrolyte regenerates the dye. Although all of the DSSC elements are equally important for a proper working principle, in this paper, we focused on one of the photoanode components—titanium dioxide.

Titanium dioxide (TiO_2_) has large numbers of polymorphs depending on pressure and temperature formation, and the most common are rutile, anatase, and brookite [14,15]. The typical DSSCs photoanodes are created with TiO_2_ in anatase form {*I*4_1_/amd, No. 141} [16,17], or with mixture of anatase and rutile {*P*4_2_/mnm, No. 136}. However, it should be considered that rutile has less efficient charge transport properties than pure anatase. Unfortunately, the main problem of the anatase form is its instability and thermal phase transformation to rutile [18]. However, it was shown that doping titania with zirconium, such as Ti_1−x_Zr_x_O_2,_ with x = 0.1, can thermally stabilize anatase, preventing its transition to rutile [14]. Unfortunately, doping with zirconium led to a widening of the energy bandgap compared to the undoped one. That is because the energy gap of ZrO_2_ is higher than 5 eV compared to the ca. 3.2 eV of anatase [14].

A number of researchers have investigated the effect of TiO_2_ doped with Zr^4+^ ions on the physicochemical properties and electrical performance of DSSCs. The most common preparation technique of nanosized TiO_2_ is the sol-gel method, but other synthetic approaches such as the solvothermal process and electrospinning have also been used [19,20,21,22]. Properly selected techniques provide different advantages, such as better size control, due to automatization of process or higher crystallinity and obtaining single-phase products. Chattopadhyay et al. synthesized spiky-shaped TiO_2_ nanocrystals doped with Zr^4+^ by solvothermal method and showed enhancement in their photoactivity up to 7.52% in comparison to the commercially used P25 (4.20%) as well as very high short-circuit photocurrent density value (21.83 mA/cm^2^) [21]. Similarly, Cavallo and coworkers synthesized undoped and TiO_2_ doped with 0.1–0.3% Zr^4+^ ions with 7% photoconversion efficiency with charge injection enhancement after introducing dopant ions [23]. Pasche, with coauthors, conducted an extensive study about the influence of annealing temperature and concentration of Zr^4+^ ions on the performance of DSSC [24]. They proved that introducing Zr^4+^ ions at a higher temperature of sintering (>400 °C) inhibits thermal degradation and boosts the overall efficiency of DSSC. One-dimensional (1D) nanofibers doped with zirconium ions were also analyzed by Mohamed et al. [25]. The enhancement of electron transfer in the TiO_2_:1%Zr nanofibers synthesized using electrospinning technique and increment of dye amount loaded on their surface was observed. It led to the obtainment of 4.51% DSSC performance value. In other studies, the increment of electron diffusion coefficient values, chemical capacitance, and overall efficiency of DSSC caused by the insertion of Zr^4+^ ions into 1D TiO_2_ have been described [22]. Data from selected publications are summarized in Table 1.

In this work, we synthesized Zr^4+^-doped TiO_2_ nanomaterials used as a photoanode component of DSSCs, leading to an unprecedented 8.63% photoconversion efficiency. The complex structural and photophysical research of semiconducting nanopowders and working electrodes with different content of Zr^4+^ ions have been made. Moreover, to characterize the role of the Zr^4+^ ions in TiO_2_ structures and to explain their influence on electronic properties of anodes, extensive theoretical calculations were performed using density functional theory (DFT) with Hubbard correction.

## 2. Materials and Methods

### 2.1. Computer Simulations

Electronic properties of the TiO_2_ crystals cannot be computed correctly by standard density functional theory (DFT). The origin of the DFT failure in transition metal oxides is associated with an inadequate description of the strong Coulomb repulsion between 3D electrons localized on metal ions [26]. In many research works, the hybrid DFT functionals or Hubbard corrections were implemented to improve the computational results [27,28].

In the presented work, structural and electronic properties of the TiO_2_ anatase crystal structure (a-TiO_2_) were calculated using the Vienna ab initio simulation package (VASP) (Version vasp.5.4.4, VASP Software GmbH, Vienna, Austria). The sw-GW basis set was used for all atoms in the system. The calculations were performed applying DFT/GGA methodology using PBE functional with Hubbard correction. The Hubbard correction was implemented into calculations as rotationally invariant LSDA+U introduced by Liechtenstein et al. [29], where the U and J as screened Coulomb and exchange parameters, respectively, are used. The Hubbard correction was applied for 3d electrons of Ti atoms, where U_Ti_ = 6 eV and J_Ti_ = 1 eV [28]. The energy cut-off for the plane-wave basis set was fixed at the value of 520 eV. The reciprocal space sampling was done with k-point Monkhorst-Pack grid 8 × 8 × 8.

**Figure 1:**
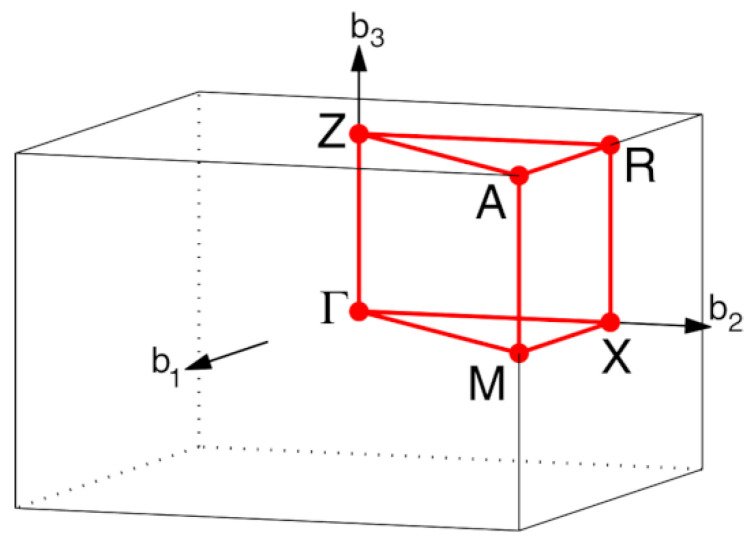
Brillouin zone path G-X-M- G-Z-R-A-Z[X-R]M-A for tetragonal crystals. Reproduced from ref. [25], with permission from Elsevier.

First, the a-TiO_2_ crystal structure was fully relaxed to obtain a minimum of total energy. In the mentioned procedure, cell parameters, volume, and atomic position have the possibility of changes. The crystals’ electronic properties were calculated in the Brillouin zone’s points specified in Figure 1, following the path G-X-M-G-Z-R-A-Z[X-R]M-A [30].

The supercell was built by repeating anatase unit cell as 2 × 2 × 1 to calculate structural and electronic properties of defected TiO_2_ crystal structures. In this case, the reciprocal space sampling was also done with k-point Monckhorst-Pack grids 8 × 8 × 8. The supercell’s electronic properties were calculated following a simple path in the Brillouin zone depicted as G-F-Q-Z-G, where F (0, 0.5, 0) and Q (0, 0.5, 0.5). In this case, all other computational parameters remained the same as described above. The TiO_2_ defected structures were built by removing one O atom or one Ti atom from the crystal supercell in anatase form. In addition, the electronic properties of the TiO_2_ structure doped by the Zr atom were calculated. The Hubbard correction was applied for 4d electrons of Zr atoms using U_Zr_ = 6 eV, J_Zr_ = 1 eV [28]. Electronic properties of the tetragonal ZrO_2_ crystal structure (t-ZrO_2_) were also calculated to check applied parameters’ correctness. The electronic properties were calculated applying Hubbard parameters U_Ti_ = U_Zr_ = 9.25 eV and J_Ti_ = J_Zr_ = 1.00 eV.

### 2.2. Materials

Titanium (IV) isopropoxide (TTIP, 97%, Sigma Aldrich, St. Louis, MO, USA), zirconium (IV) n-propoxide (ZrP, solution 70 wt% in 1-propanol, Sigma Aldrich, St. Louis, MO, USA), acetic acid (p.a., POCh, Gliwice, Poland), nitric acid (p.a., POCh, Gliwice, Poland), and freshly distilled before synthesis 2-propanol (99.8%, WITKO, Poland) were used for synthesis. Titanium (IV) chloride (99.9%, Sigma Aldrich, St. Louis, MO, USA), anhydrous ethanol (p.a., POCh, Gliwice, Poland), acetone (p.a., PPH Stanlab, Poland), cis-Bis(isothiocyanato)bis(2,2′-bipyridyl-4,4′-dicarboxylato)ruthenium(II) (N3 dye, 95% NMR, Sigma Aldrich, St. Louis, MO, USA), chloroplatinic acid (99.9% Merck, Darmstadt, Germany), ammonia water solution (25%, p.a. POCh, Gliwice, Poland), α-Terpineol (96%, Sigma-Aldrich, St. Louis, MO, USA), and ethylcellulose (p.a., Sigma-Aldrich St. Louis, MO, USA) were applied, among others, for cleaning TCO glass and preparing 10^−4^ M dye’s solution, titania’s pastes, protective coating of TiO_2_, Pt electrodes and the dye desorption process. The electrolyte mixture consisted of 0.03 M iodine (Sigma Aldrich, St. Louis, MO, USA), 0.6 M 1-propyl-3-methyl-imidazolium iodide (Sigma Aldrich, St. Louis, MO, USA), 0.1 M guanidine thiocyanate (Sigma Aldrich, St. Louis, MO, USA), and 0.5 M 4-tert-butylpiridine (Sigma Aldrich, St. Louis, MO, USA) in acetonitrile (p.a., POCh, Gliwice, Poland). TCO22-7 FTO glass (Solaronix, Aubonne, Switzerland) and ionomeric foil Meltonic (Solaronix, Aubonne, Switzerland) were used as substrates and sealing material, respectively. P25 Aeroxide (Evonik, Essen, Germany) was used as a reference titanium dioxide nanopowder. Deionized water was employed at every step of the experiment.

### 2.3. Preparation Method

The synthesis of materials was performed applying a modified version of the sol-gel method [31]. Generally, 0.592 mL of TTIP (and 9.06 µL, 27.73 µL or 47.18 µL of ZrP were designated as 1.2%, 3.7%, and 5.6% of Zr^4+^ ions, respectively, in the case of doped TiO_2_) was added to 200 mL of 2-propanol in a two-neck round bottom flask and mixed for 15 min. Vigorous stirring occurred throughout the whole process. Subsequently, the mixture of 10 mM acetic acid in 100 mL of isopropanol was added dropwise for another 40 min. In the next step, 30 mL of deionized water was introduced to the as-prepared solution with a 0.5 mL/min flow rate by a syringe pump (MEDIMA S200) (Medima, Warszawa, Poland). Afterward, 0.4 mL of nitric acid was injected, and the mixture was heated under reflux for 75 min. Finally, additional 7 mL of deionized water was added, and the reaction was refluxed for 24 h. The as-obtained colloidal solution was filtered under reduced pressure (Whatman NL17 Polyamide Membrane Filters 0.45 µm) (Whatman plc part of Cytiva, Marlborough, MA, USA), washed with ethanol several times, and dried overnight at 60 °C. Samples were divided into three parts. One of them was analyzed by FT-IR and TGA-DTA techniques, the second was annealed at 450 °C for 2 h with a 7.5 °C/min ramp rate for further analyses, and the last one was used for viscous paste preparation.

### 2.4. DSSC Fabrication

Before the cell’s fabrication, FTO substrates were sonicated for 30 min in a 1:1 (*v*/*v*) acetone and ethanol mixture. The viscous paste was prepared by mixing particulate samples with other components based on a ratio: 1 g of nanopowder, 1 mL of acetic acid, and 20 mL of ethanol, and it was kept in an ultrasound bath for 3 h. Afterward, a solution of 1.5 g of ethylcellulose, 10 mL of α-terpineol, and 13.5 g of ethanol was added to a nanopowders colloid. The above mixture was kept in an ultrasound bath for 1 h and finally stirred overnight [32]. Finally, the excess of ethanol was evaporated, and paste was spread on FTO glass via the “doctor blade” technique with scotch tape as a template (62.5 µm of thickness). Next, FTO substrates with TiO_2_ layers were calcined in the air for 2 h at 450 °C with a ramp rate of 7.5 °C/min. As-prepared photoelectrodes were immersed in a 40 mM aqueous solution of TiCl_4_ for 1 h at 70 °C. Subsequently, working electrodes were washed with water and ethanol, dried in hot air, and again annealed for 30 min at 450 °C. Finally, photoanodes were sensitized overnight with a 10^−4^ M N3 dye solution in a staining chamber. Counter electrodes were prepared by wiping predrilled FTO glass with a tissue soaked in H_2_PtCl_6_ ethanolic solution (23 g/L of Pt) and then annealed at 450 °C for 30 min. Finally, photoelectrodes were combined with a 25 μm thick ionomeric foil as a sealant and a spacer. The electrolyte was injected through two holes predrilled in the photocathodes, and the devices were finally sealed by hot melted foil and microscopic slide. The typical active area of the cells presented in this work was approximately 0.125 cm^2^.

### 2.5. Dye Loading Determination

Additionally, the particular working electrodes with an active area of about 3 cm^2^ were prepared to determine the dye amount adsorbed on the TiO_2_ film surface. These photoanodes were immersed in a 2 M ammonia solution in ethanol for 30 min to investigate the number of dye molecules adsorbed on the TiO_2_ films. Afterward, the desorbed dye concentration in the obtained solution was examined using UV-Vis measurement at 310 nm based on the calibration curve. The above procedure has been made for five electrodes of each type, and the presented results are the average of these five measurements.

### 2.6. Characterization

The structural analyses were employed using the X-ray diffraction (XRD) examination on the D8 Advance diffractometer (Bruker, Billerica, MA, USA) with λ = 0.15406 nm Cu Kα radiation. The reference patterns of anatase, rutile, brookite, and tin oxide were taken from the International Centre for Diffraction Data (ICDD). Scherrer’s equation was applied to determine the crystallites’ size [33]:

(1) $$D_{hkl}=\frac{K\lambda }{\beta_{hkl}cos\theta },$$

where *D_hkl_* is a crystallite size, *K* is a shape factor equal to 0.9, *λ* is a radiation wavelength (0.15406 nm), *β_hkl_* is the line broadening half the maximum intensity (FWHM) in radians, and *θ* is the Bragg angle. While the lattice parameters (*a* and *c*) were calculated based on the equation for tetragonal type of phase [34,35]:

(2) $$\frac{1}{d^{2}}=\frac{h^{2}+k^{2}}{a^{2}}+\frac{l^{2}}{c^{2}},$$

where *d* is the interplanar spacing:

(3) $$d=\frac{\lambda }{2\;sin\theta },$$

For calculation, the (004) and (200) reflexes were used.

Transmission electron microscopy (TEM) images were recorded on a Hitachi HT7700 microscope (Hitachi, Tokyo, Japan), operating at an accelerating voltage of 100 kV. Samples were dispersed in ethanol and sonicated for 5 min, then deposited at copper grids coated with carbon. Scanning electron microscopy (SEM) images and energy-dispersive X-ray spectroscopy (EDS) were taken on FEI Quanta FEG 250 (FEI Company, Hillsboro, OR, USA) at 30 kV. The concentration of dopant ions in TiO_2_ nanoparticles was examined via X-ray fluorescence spectroscopy (XRF) on MiniPal2 apparatus (PANalytical B.V., Almelo, The Netherlands). The calibration curve was prepared by mixing ZrO_2_ and P25 with the increasing amount of the latter. Subsequently, all powders were ground in a ball mill (Mixer/Mill 8000M, Spex, New York, NY, USA) equipped with a zirconia ceramic vial set for 30 min. Fourier transforms infrared (FTIR) spectra were registered on an IFS-66/s spectrometer (Bruker, Billerica, MA, USA) using KBr powder as a dilutant. The samples’ structure was also investigated using an inVia Raman microscope (Renishaw plc, Wotton-under-Edge, UK) with an excitation beam at 514 nm. The phonon lifetimes were calculated for E_g_ mode, based on the relation of the energy-time uncertainty:

(4) $$\frac{1}{\tau }=\frac{\Delta E}{\hbar }=2\pi c\Gamma ,$$

where Δ*E* is uncertainty in phonon mode’s energy, ℏ indicates Planck’s constant, *c* is the speed of light, and *Γ* is the FWHM of the Raman peaks (cm^−1^). The bandgap’s width was determined via diffuse-reflectance spectroscopy (DRS) on Cary 5000 spectrometer (Varian, Palo Alto, CA, USA) equipped with a 100 mm diameter integrating sphere and using a BaSO_4_ powder as a reference—total reflectance material. The bandgap value was established by plotting the Tauc Equation:

(5) $${\left(\alpha h\upsilon \right)}^{n}=A\left(h\upsilon -E_{bg}\right),$$

where *hυ* is the energy of an incident photon, *α* is absorption coefficient, *n* determines electronic transitions linked to the absorption processes (*n* = ½ allowed indirect), *A* describes a constant, and the *E_bg_* is a bandgap.

The N_2_ adsorption-desorption isotherms at 77 K curves were recorded by a Nova 1200e sorptometer (Quantachrome Instruments, Boynton Beach, FL, USA). Specific surface area was determined using Brunauer-Emmett-Teller (BET) method and simultaneously, the average pore volumes (V_p_) and diameters (S_p_) were calculated using a Barrett–Joyner–Halenda (BJH) equation based on the desorption branch. The electron paramagnetic resonance (EPR) spectra were recorded at 77 K. They were run on an X-band (~8.9 GHz) CW-EPR SE/X-2547 spectrometer (Radiopan, Poznań, Poland) with a reflection type resonator and 100 kHz modulation of the magnetic field. X-ray Photoelectron Spectroscopy (XPS) was carried out at ultra-high vacuum (<2 × 10^−8^ mbar) on spectrometer SPECS Surface Nano Analysis GmbH (Berlin, Germany) to determine the surface bonding and atomic concentration. The binding energies of all peaks were corrected and shifted concerning the C 1s signal, defined as an adventitious carbon with a set value of 284.8 eV, to yield meaningful results. Ultraviolet Photoelectron Spectroscopy (UPS) measurements were carried out on the same equipment as XPS measurements with UVS 10/35 light source and He I 21.2 eV ionization source. The Jupiter STA 449 F3 (Netzsch GmbH, Selb, Germany) experimental equipment was applied for thermogravimetric analysis and developed in the air atmosphere and 30–1000 °C temperature range (10 °C/min). The amount of dye adsorbed on titania films was determined via the UV-Vis technique on a Cary 50 (Varian, Palo Alto, CA, USA) spectrometer. The current density and photovoltage characteristics (J-V), as well as electrochemical impedance spectroscopy (EIS), was conducted on Gamry Interface 1000 Potentiostat/Galvanostat/ZRA (Gamry Instruments, Warminster, PA, USA) with Sun 2000 Solar Simulator (ABET Technologies, Inc., Milford, MA, USA) light source under the simulated AM 1.5G (100 mW/cm^2^) conditions. The efficiency (*η*) and fill factor (*FF*) values were calculated based on the following Equations:

(6) $$\eta =\frac{P_{MAX}}{P_{IN}}=\frac{V_{OC}\times J_{SC}\times FF}{P_{IN}}\times 100\%,$$

(7) $$FF=\frac{J_{MAX}\times V_{MAX}}{J_{SC}\times V_{OC}}\times 100\%,$$

where *P_MAX_* is maximum device power, *V_OC_* is open circuit photovoltage, *J_SC_* is short circuit photocurrent density, *FF* is fill factor, and *P_IN_* is the power of incident light.

Electron lifetime (*τ*) have been calculated using the frequency (*f*) of the maximum point at the mid-frequency arc of the Bode plot according to the Equation:

(8) $$\tau ={\left(2\pi f\right)}^{-1}.$$

Incident photon-to-current efficiency (IPCE) measurements were developed on Bentham PVE300 EQE/IPCE (Bentham Instruments Limited, Reading Berkshire, UK). The IPCE is a conversion ratio between the number of charge carriers collected to the cell to the number of photons of a given energy.

## 3. Results and Discussion

### 3.1. Theoretical Calculations

#### 3.1.1. Structural Properties

The a-TiO_2_ and t-ZrO_2_ crystal structures were relaxed applying DFT/PBE+U method, and the obtained unit cell parameters are presented in Table 2. These results show that the DFT/PBE+U method with U_Ti_ = 6 eV and J_Ti_ = 1 eV reproduces the structure of the a-TiO_2_ acceptably. One can see that the modeled unit cell of the a-TiO_2_ is slightly larger than the experimentally studied one. However, the deviation of the lattice parameters compared to experimentally obtained data is less than 2.1%. It allows us to conclude that the obtained structure can be used to calculate the electronic properties of the a-TiO_2_ crystal. Moreover, the t-ZrO_2_ crystal unit cell’s side lengths are also overestimated using the DFT/PBE + U method. It should be noted that all computational parameters were used the same as for a-TiO_2._ In the case of the t-ZrO_2_, the deviation of obtained lattice parameters from experimental data measured in temperature 293 K is less than 2.3% and is less than 1% for the ones investigated in temperature equal to 1543 K [36,37]. One can conclude that the quantum-chemical calculations based on the DFT method with chosen Hubbard parameter reproduce the experimental structure of studied crystals. Additionally, comparing total energies per atom of both structures, it can be said that the t-ZrO_2_ is more stable than the structure of the a-TiO_2_. It is caused by the fact that the Zr-O interaction is stronger than the Ti-O interplay.

In the a-TiO_2_ crystal structure, six O atoms creating octahedron (see Figure S1 at Supplementary Materials) surround each Ti atom. The Ti-O bonds along c direction are longer than those lying in the ab plane, and they are equal to 1.966 Å and 1.937 Å, respectively [41]. Both calculated Ti-O bonds are longer than the experimental ones, and they are equal to 2.001 Å and 1.967 Å, respectively. The spicier of the octahedron is slightly longer. Although the modeled Ti-O bonds are more extended than the experimental results, the O-Ti-O angles are almost identical. Experimental angles are equal to 102′38 and 92′60, and the modeled ones are equal to 102′23 and 92′57. Obtained results confirm that performed calculations well reproduce the structure of the a-TiO_2_.

In the t-ZrO_2,_ each zirconium atom maintains its eight oxygen coordination: four oxygen atoms at a distance of ~2.10 Å and four at a distance of ~2.30 Å [42]. Performed calculations give a length of these bounds equal to 2.18 Å and 2.32 Å, respectively. It can be concluded, comparing the theoretical data with the experimental ones, that the DFT method with the adopted Hubbard parameters well reflects the structure of a-TiO_2_ and t-ZrO_2_ crystals.

To investigate defected structures with vacancies or dopants not exceeding a few percent of the tested material’s composition, the supercell of the a-TiO_2_ was built. The new supercell was constructed by 2 × 2 × 1 repetition of the a-TiO_2_ unit cell. The constructed a-TiO_2_ supercell (2 × 2 × 1 TiO_2_) was also relaxed applying the DFT/PBE+U method (U_Ti_ = 6eV, and J_Ti_ = 1 eV). Analyzing data collected in Table 2 shows that the supercell parameters are equal to the a-TiO_2_ unit cell parameters. The total energy per atom for the unit cell of the a-TiO_2_ and supercell is also the same. It means that the constructed supercell can be used for further calculations.

The oxygen v(O) and titanium v(Ti) vacancies were introduced into the 2 × 2 × 1 TiO_2_ supercell. One oxygen or titanium atom was removed from the structure giving a 6% crystal defect in both situations. Oxygen vacancies (2 × 2 × 1 TiO_2_ v(O), see Table 2) practically do not change unit cell lengths compare to the stoichiometric crystal (2 × 2 × 1 TiO_2_). The changes are more significant for the titanium vacancies (2 × 2 × 1 TiO_2_ v(Ti)). Consequently, the volume of the unit cell diminishes with the existence of the v(Ti). One can also see that the total energy per atom increases with oxygen and titanium vacancies compared to the stoichiometric a-TiO_2_ crystal. It means that the defective structure is less stable than the stoichiometric crystal. However, it is worth noticing that oxygen vacancies stabilize the structure more than titanium vacancies.

The 2 × 2 × 1 TiO_2_ structure was doped by Zr^4+^ ions located in interstitial position (2 × 2 × 1 TiO_2_ + Zr) or substituting the Ti atom (2 × 2 × 1 TiO_2_Zr). A total energy per atom is lower in the case of substituting than an interstitial Zr atom. It should also be noted that the 2 × 2 × 1 TiO_2_ structure doped by replacing Zr atom (2 × 2 × 1 TiO_2_Zr) is more stable than the pure a-TiO_2_ crystal structure, but the energy of the 2 × 2 × 1 TiO_2_ + Zr is comparable to the total energy of the 2 × 2 × 1 TiO_2_. It allows us to conclude that about 6% of the Zr dopants stabilizes the a-TiO_2_ crystal structure. The Zr dopants do not change the parameters of the a-TiO_2_ crystal unit cell significantly. However, in both cases, the unit cell increases compared to the 2 × 2 × 1 TiO_2_ crystal structure. It is caused by an increase in the sides a and b of the unit cell. The length of side c remains unchanged in both instances of the substitution of the Zr atom.

The 2 × 2 × 1 TiO_2_ crystal structures doped by Zr atoms were also modeled with oxygen vacancies. The v(O) was created close to the Zr atom and far from the Zr atom. They have been marked as 2 × 2 × 1 TiO_2_Zr v(O), 2 × 2 × 1 TiO_2_+Zr v(O), and 2 × 2 × 1 TiO_2_Zr v(O)far, 2 × 2 × 1 TiO_2_+Zr v(O)far, respectively. The oxygen vacancies present in Zr doped a-TiO_2_ structure do not stabilize the crystal more compared to the 2 × 2 × 1 TiO_2_Zr and 2 × 2 × 1 TiO_2_ + Zr structures, respectively. One can conclude that as was observed for the virgin a-TiO_2_, vacancies destabilize the crystals. However, structures 2 × 2 × 1 TiO_2_Zr v(O) and 2 × 2 × 1 TiO_2_Zr v(O) possess total energy per atom than the energy of the non-doped a-TiO_2_ structure. The Zr doping of the a-TiO_2_ v(Ti) structure was not modeled due to the high total energy per atom of the a-TiO_2_ structure with Ti vacancies.

Analyzing performed calculations, it can be concluded that the most probable are a-TiO_2_ structures doped by Zr in substituting position with oxygen vacancies far from Zr atom. It means that the Zr atoms should be observed in the Zr^4+^ state, but the titanium atoms should be observed in Ti^4+^ and Ti^3+^ state.

#### 3.1.2. Electronic Properties of a-TiO_2_ and t-ZrO_2_

One of the most essential and common parameters representing the properties of crystals is their bandgap. The calculated energy gap compared with experimentally obtained data can check the calculation’s correctness method. Performed calculations of electron properties of the a-TiO_2_ crystal proved that the Hubbard correction parameters are not universal. The optimization procedure of studied structures, giving good results was performed with parameters U_Ti_ = U_Zr_ =6 eV and J_Ti_ = J_Zr_ = 1 eV. Unfortunately, the bandgap value calculated with that parameter is underestimated, offering the same values as the conventional DFT method (in the case of a-TiO_2_ E_g_ ∼ 2.2 eV and t-ZrO_2_ E_g_ ∼ 3.8 eV). Therefore, the a-TiO_2_ crystal structure electronic properties were calculated with Hubbard parameters U_Ti_ = 9, 9.1, 9.25, 9.50, 9.75, and with J_Ti_ = 1. However, the correct energy gap compared with the experiment was obtained using U_Ti_ = 9.25 eV and J_Ti_ = 1. It was also proved that the same parameter works well for calculations performed for t-ZrO_2_.

**Figure 2:**
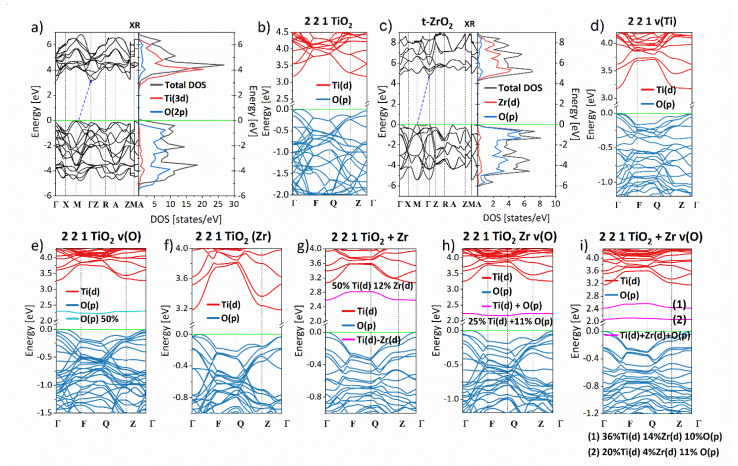
Energy band structure calculated by DFT/PBE+U method for: a-TiO_2_ crystal in primitive unit cell representation (**a**), a-TiO_2_ supercell (2 × 2 × 1 TiO_2_) (**b**), t-ZrO_2_ crystal (**c**), defected a-TiO_2_ crystal with titanium vacancies (**d**) and oxygen vacancies (**e**), a-TiO_2_ supercell doped by Zr substituting Ti atom (**f**) and in interstitial position (**g**), a-TiO_2_ doped by Zr atoms substituting Ti atoms (**h**) and in interstitial position (**i**) additionally possessing oxygen vacancies.

The energy band structure and the electron density of states (DOS), calculated for the a-TiO_2_ using the parameters mentioned above, is presented in Figure 2a,b. One may see that the a-TiO_2_ is an indirect semiconductor with a calculated energy gap equal to 3.16 eV. It is in good agreement with an experimentally measured energy bandgap of a-TiO_2_ equal to 3.20 eV. The a-TiO_2_ is a typical metal oxide of the form AB_2_ for which O 2p electrons create the valence band, and d-Ti states construct the conduction band.

Electronic properties of the (2 × 2 × 1 TiO_2_) supercell were also calculated to check the correctness of the chosen model, and the obtained data are presented in Figure 2b. Comparing two energy band structures calculated for the a-TiO_2_ and the 2 × 2 × 1 TiO_2_ structure, one can conclude that the extension of the primitive unit cell to the supercell does not change its electronic parameters. It means that the proposed supercell can reproduce the character of the a-TiO_2_ crystal structure.

The energy band structure was also calculated for the t-ZrO_2_ crystal using the same computational parameters as implemented for the a-TiO_2_. The obtained results are presented in Figure 2c. The t-ZrO_2_ crystal valence band, as in the case of the a-TiO_2_ crystal, is built with oxygen states and a conduction band with zirconium states. The t-ZrO_2_ is also an indirect semiconductor with a bandgap equal to 4.83 eV that is in satisfactory agreement compared to the experimentally measured energy gap equal to 5.0 eV [14]. Calculations performed for a-TiO_2_ and t-ZrO_2_ conclude that the implemented DFT quantum-chemical method augmented by Hubbard correction with U and J parameters specially selected for relaxation of the crystals and their electronic properties calculations can be used to study doped and defected a-TiO_2_.

The 2 × 2 × 1 TiO_2_ structure was used to calculate properties of the defected a-TiO_2_ crystal. Two kinds of defects were constructed: oxygen vacancies v(O) and titanium vacancies v(Ti). Calculated energy band structures are presented in Figure 2d–e. Comparing the energy band structure obtained for 2 × 2 × 1 TiO_2_ (see Figure 2b) and the ones presented in Figure 2d–e, it may be seen that vacancies do not change the shape of the valence bad. However, both vacancies change the bottom of the conduction band. Comparing these data to results obtained for virgin a-TiO_2_ structure, one can see that low-lying conductionbands of defected structures are less dispersed. They do not cross one other. Additionally, the v(O) creates an additional occupied energy band located in the bandgap region. This band is constructed mainly by oxygen states, but its DOS intensity is very low.

Electronic properties of the Zr^4+^-doped a-TiO_2_ crystal structure were also calculated. In one case, the Zr atoms replace the Ti atoms, but in the second case, the Zr atoms are in an interstitial position. The amount of dopants is equal to 6%. The energy band structures calculated for the Zr^4+^-doped crystals are presented in Figure 2f,g. The energy band structure calculated for the 2 × 2 × 1 TiO_2_Zr looks like the structure calculated for a-TiO_2_ with titanium vacancies. The deeply lying zirconium states do not change the electronic structure of the a-TiO_2_. The situation is different when the Zr is in the interstitial position of the a-TiO_2_ crystal. Here titanium and zirconium electrons create an additional energy band located below the bottom of the conduction band. The DOS intensity of the formed energy band is very low.

The electronic band structures calculated for a-TiO_2_ crystal doped by Zr^4+^ ions and oxygen vacancies are presented in Figure 2h,i. Obtained energy dependencies are a superposition of the energy bands illustrated in Figure 2d–g. Crystals with oxygen vacancies and doped by Zr atoms retain the energy band structure of the Zr^4+^ ion-doped structures and energy band structure of the a-TiO_2_ defected by oxygen vacancies. Also, the DOS intensities of additional energy bands created in the energy gap range are very low. One can conclude that they will not be seen in the experimentally obtained value of an energy gap.

In Table 3, the energy gap values calculated for all evaluated crystal structures are collected. One can see that the Zr atom substituting Ti atom does not change the energy gap of the a-TiO_2_ crystal. The Zr atom in the interstitial position decreases the energy gap value of the a-TiO_2_ crystal. Oxygen vacancies increase the energy gap no matter where they are located, far or close to the Zr atom.

**Figure 3:**
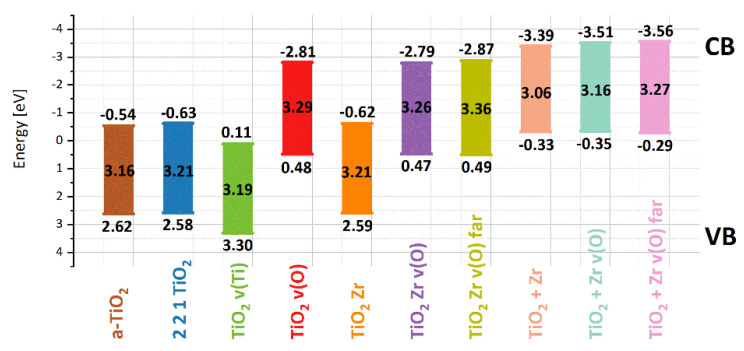
Valence band maximum and conductionband minimum for structures based on a-TiO_2_ crystals structure modified by dopants and vacancies calculated by DFT/PBE+U method.

From Figure 3, one can see that the oxygen vacancies decrease the value of the conduction band minimum and valence band maximum level. The v(O) in the TiO_2_ structure increases the mentioned energy levels, but the anatase structure with Ti vacancies is the least likely from total energy analysis.

### 3.2. Experimental Determination

#### 3.2.1. Structure and Morphology

**Figure 4:**
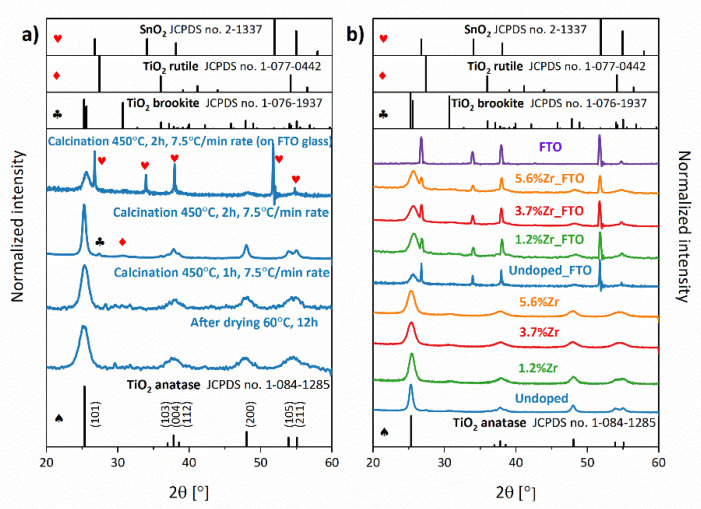
XRD patterns of bare TiO_2_ annealed at different conditions (**a**), and Zr^4+^-doped TiO_2_ in the form of nanopowders and as working electrodes (on FTO substrate) (**b**).

Figure 4 shows the XRD spectra of the TiO_2_ matrices obtained at varied temperatures (a) and TiO_2_ doped with different concentrations of Zr^4+^ ions in the form of powders and as the layers deposited on FTO glass after annealing at 450 °C for 2 h (b). The content of Zr^4+^ ions (1.2, 3.7, and 5.6%) was determined by the XRF technique (see Figure S2). It can be noted that all of the samples exhibit mainly anatase structures (ICCD 1-084-1285). The XRD patterns of materials deposited on the FTO substrate show the sharp and narrow reflexes of the underneath SnO_2_ conductive layer (ICCD 2-1337). In Figure 4a, it may be seen that the nanocrystals’ mean size increases with an increase of treatment temperature, ranging from 4.89 up to 6.44 nm for TiO_2_ dried at 60 °C for 12 h and TiO_2_ annealed at 450 °C for 1 h, respectively. It is worth mentioning that annealing time is also crucial in nanocrystals’ growing process, and time elongation from 1 to 2 h caused a further rise of crystal size from 6.44 to 12.85 nm. As shown in Figure 4a, anatase reflexes, especially in dried TiO_2_, are broadened. Among other things, it indicates that crystal surface on the grain interface may contain defects [43]. The presence of defects caused an increase of strains in the lattice and prevented the growth of crystals. Therefore, an extension of the annealing process time may cause a diffusion phenomenon and disappearance of grain boundaries, which leads to the coalescence of crystals into the bigger one [44]. Finally, a narrowing of the reflections in diffractograms was observed.

Moreover, if dried at 60 °C, TiO_2_ is directly used for a viscous paste preparation and calcinated under the same conditions (450 °C, 2 h). After deposition on FTO glass, the crystal size is diminished from 12.85 to 7.61 nm. This can indicate that when the sample is being annealed on FTO substrate, the SnO_2_ can migrate into the TiO_2_ structure and inhibit the nanoparticles’ growth [45]. A similar situation may be observed when Zr^4+^ ions are doped into the TiO_2_ crystal site (Figure 4b). Aside from nanoparticles’ size-changing during the time elongation of the calcination process, the phase structure of investigated samples has also changed. Dried TiO_2_ matrices annealed at 450 °C for 1 h are single-phase products, whereas the rutile and brookite phases appeared in the titania nanoparticles calcinated for 2 h. However, introducing Zr^4+^ ions into TiO_2_ or annealing TiO_2_ paste on FTO glass caused inhibition of the anatase to rutile phase transformation [46,47,48].

Introducing 6% Zr^4+^ ions, which have a larger ionic radius than Ti^4+^ ions (0.72 Å and 0.69 Å, correspondingly), do not change the lattice parameters of TiO_2_ remarkably, referring to the theoretical calculations. Compared to the calculated unit cell parameters (see Table 2), a similar situation was observed in the experimental results (see Table 4). Nonetheless, it may be concluded that Zr^4+^ ions were successfully substituting the Ti^4+^ ions in the presented materials. This statement may be supported by the differences between the total energy per atom (see Table 2) for substitutional (2 × 2 × 1 TiO_2_Zr) and interstitial Zr^4+^ ions (2 × 2 × 1 TiO_2_ + Zr) arrangements, which are −8.78 and −8.70 eV, respectively, and suggests higher stability of the former form.

Slight differences in cell parameters between the experiment and theoretical calculations result from the fact that the temperature of 0K was assumed in the calculations.

**Figure 5:**
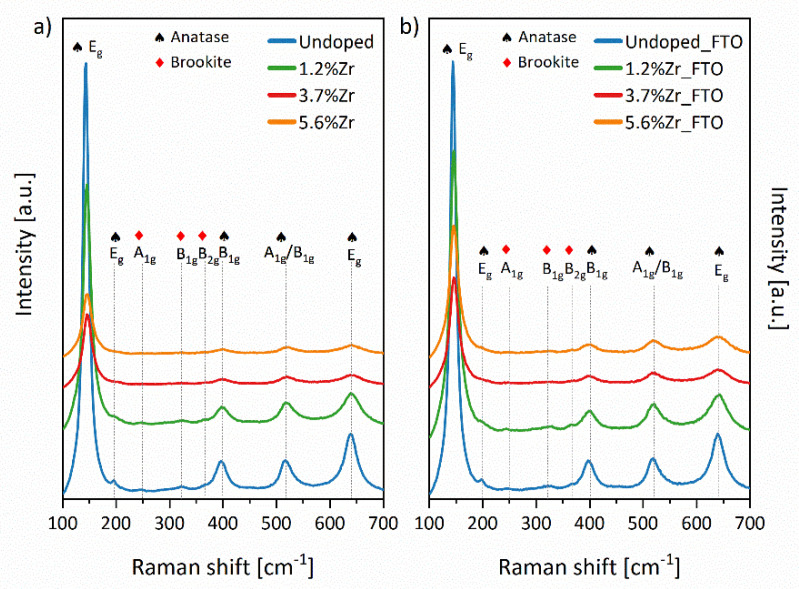
Raman spectra of TiO_2_ nanopowders (**a**) and FTO nanoparticles (**b**) annealed at 450 °C for 2 h.

Raman spectra of TiO_2_ nanopowders and TiO_2_:Zr_FTO were recorded and are presented in Figure 5a,b. Intense peaks at about 145, 196, 398, 519, and 639 cm^−1^ were observed for the corresponding anatase modes: E_g(1)_, E_g(2)_, B_1g(1)_, A_1g_/B_1g(2)_, and E_g(3)_, respectively. Crystal lattice vibrations A_1g_ and B_1g(2)_ are superimposed in the plot at 519 cm^−1^ and may only be separated at low-temperature measurements [49]. The additional low-intensity bands at about 245, 323, and 368 cm^−1^ may be observed and assigned to the A_1g_, B_1g_, and B_2g_ of the brookite structure [50]. The low-intensity and broad peaks mentioned above may be explained by the high structural disorder and brookite phase partial amorphization [51]. The presence of brookite peaks and no additional phase are in good agreement with the XRD results described above.

The linewidth and peak position of Raman spectra may be influenced by many factors, e.g., phonon confinement, anharmonic effects, crystals size, as well as temperature or crystal defects, and strains of lattice sites [52,53,54,55,56,57]. The detailed Raman data presented in Table S1 (based on the bands’ deconvolution using Lorentz fitting) has been compiled to distinguish particular samples. It should be noted that after loading of dopant ions, the E_g_ mode scattering intensities in Raman spectra decreased. Komaraiah and co-workers also observed similar effects and described it as the lattice periodicity changes and crystal symmetry translation in the long-range [34]. It might be induced by defects or distortion in the crystal lattice. Furthermore, the E_g_ mode blue shift of about 2–3 cm^−1^ is also observed and may be linked to the minimalizing of nanoparticles’ size.

Moreover, the phonon lifetime was also calculated based on E_g_ mode at 144 cm^−1^ (see Figure S3 and Table S2) and indicated the decline from 0.390 to 0.263 ps and 0.260 ps incrementing Zr species in the case of nanoparticles scratched off from the FTO substrates (called further as FTO nanoparticles) and nanopowders, respectively. This observation may support the statement about imperfections in the crystal lattice of obtained TiO_2_ materials [34].

**Figure 6:**
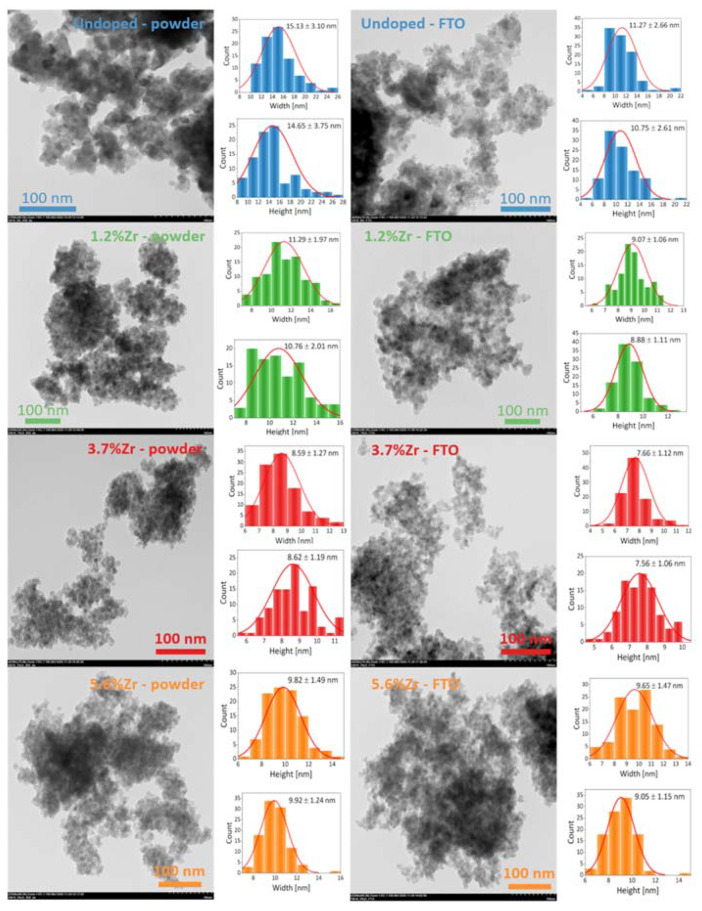
TEM images of undoped TiO_2_ and Zr-doped nanopowders with distribution histograms annealed at 450 °C for 2 h (**left column**), and FTO nanoparticles (**right column**).

The size and morphology of titanium dioxide nanoparticles calcinated at 450 °C for 2 h were visualized by TEM images. Figure 6 shows pristine TiO_2_ and Zr^4+^-doped TiO_2_ nanopowders and FTO nanoparticles with histogram distribution for width and height dimensions of particulate particles. Undoubtedly, there is a correlation between TEM and XRD results because of the particle sizes’ similar tendency. It can be seen that all samples tend to the aggregation, even though the sonication treatment was used for FTO nanoparticles. Furthermore, prepared nanoparticles had a very regular, spherical shape with a narrow size distribution. Simultaneously, a similar observation was made in the SEM images, presented in Figure S4. The hydroxyl group present on the TiO_2_ surface and the size to volume ratio may explain particles’ aggregation observed in both microscopic techniques [58,59].

**Figure 7:**
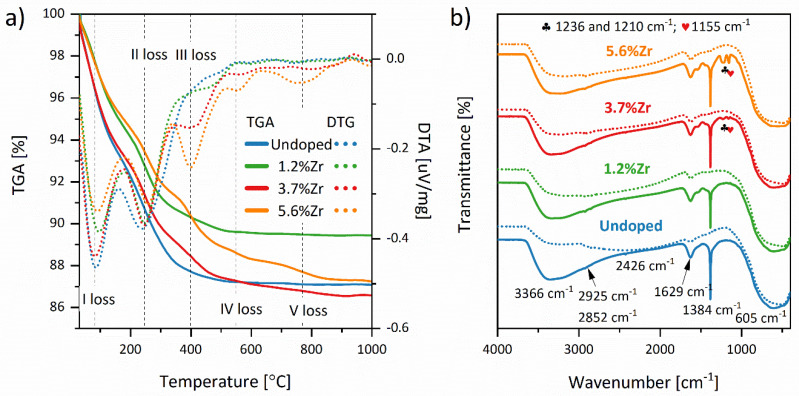
TGA and DTG thermograms of TiO_2_ nanoparticles after drying at 60 °C (**a**), and FTIR spectra of materials dried at 60 °C (straight line) and annealed at 450 °C for 2 h (dotted line) (**b**).

The thermogravimetric (TGA) and derivative thermogravimetry (DTG) analyses of Zr^4+^ ions doped TiO_2_ have been performed, and the results are presented in Figure 7a. The first weight loss in the range of 83–96 °C is a result of the dehydration processes and/or escape of CO_2_ molecules trapped in the materials’ pores. In the range of 241–258 °C, the second weight loss may be assigned to the decomposition and/or oxidation of post-synthetic organic residues in the material. The third weight loss (396–550 °C) may be linked to the formation of defective titanium dioxide with oxygen vacancies. The other perceptible drop in TGA, observed in the materials doped with 3.7 and 5.6% of Zr^4+^ ions (>550 °C), may be related to the other ZrO_2_ phase transformations and composition of the lattice defects.

To better understand the interaction between the synthesis substrates, FTIR spectroscopy was carried out. In Figure 7b are presented FTIR spectra of samples dried at 60 °C (straight line) and after calcination at elevated temperature (dotted line). Metalloxane bondings M–O–M’ (M and M’ = Zr, Ti) at about 475–625 cm^−1^ are formed via M–OR (M = Ti, Zr, and R = isopropoxide, n-propoxide) and water hydrolysis, followed by a condensation process of creating in situ M–OH and M–OR (or other M–OH) groups [60,61,62]. The narrow peak at 1385 cm^−1^ corresponds to the σ(C–H) bonding from alkoxy groups, and it is being overlapped with C–O stretching vibration caused by alkoxy residue [60,63]. The isopropanol and n-propanol residues’ bands at about 2854–2974 cm^−1^ may be attributed to the –CH_2_ and –CH_3_ symmetrical and asymmetrical stretching bonds [64]. A peak at 1628 and in the range of 3200–3364 cm^−1^ is linked to the –OH groups bending and stretching bonds, respectively [65]. Their intensities decreased after calcination at 450 °C, whereas the peak at 2426 cm^−1^ corresponding with atmospheric CO_2_ (which can be adsorbed in the material’s pores) is not observed after high-temperature treatment [66]. The more visible differences between the spectra of dried and annealed materials may be observed in the fingerprint region. It is noted that an inconsiderable peak at 1160 cm^−1^ is linked to the stretching C–O vibrations emerging due to the RCO–M bonding [60,67]. Two peaks at about 1440 and 1540 cm^−1^ may point out the acetate group complexation with M ions and are assigned to asymmetric ν_asym_ (COO) and symmetric ν_sym_ (COO) stretching bonds, correspondingly [68]. The frequency separation between these two bands is equal to Δν = 100 cm^−1^ and suggests acetate’s coordination in bidentate geometry. That means that acetate ions may create bidentate and bridging ligands with both metal ions (Zr and/or Ti) [69,70]. It is worth noticing that with Zr^4+^ ions’ increasing content, the band at about 1228–1233 cm^−1^ may be observed and is no longer visible after the material calcination. It may be correlated with the acetate group of ester-isopropyl acetate or n-propyl acetate created after condensation of M-O-M’ species [71]. Bands at 1769 cm^−1^ assigned to the carbonyl moieties are also indicated in the spectra recorded after the drying process. The more detailed FTIR data were collected in Table S3.

**Figure 8:**
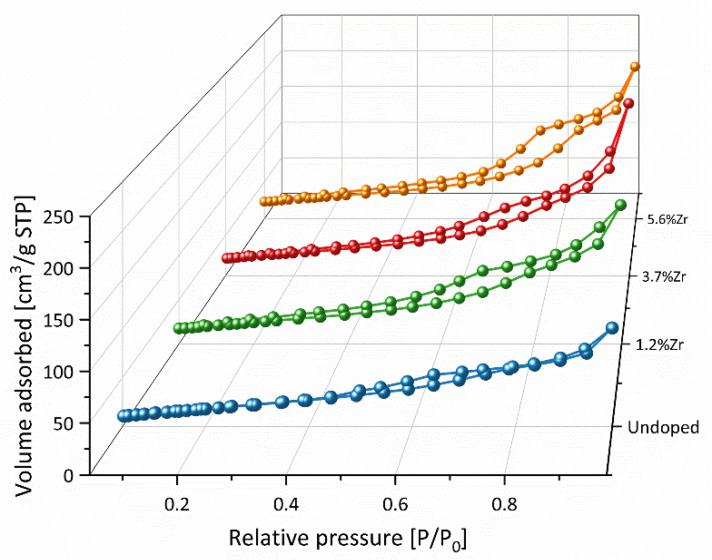
Nitrogen adsorption-desorption isotherm curves of nanopowders annealed at 450 °C for 2 h.

Nitrogen adsorption-desorption isotherm curves of nanopowders annealed at 450 °C for 2 h, presented in Figure 8, have been performed to investigate the porosity type and specific surface area. Based on IUPAC classification, the type IVa isotherm with H2a hysteresis loops may be distinguished for all the materials presented [72]. Initially, between 0 and 0.6 relative pressure, a gradual increment of the adsorbed volume may be observed, then sharp triangular hysteresis appeared. It is worth noticing that doping Zr^4+^ ions into TiO_2_ matrices leads to surface area increment from 69.4 to 132.9 m^2^/g for pristine TiO_2_ and 5.6% of Zr^4+^ doped species, respectively (see Table 5). It may be linked to the previous XRD and TEM results because the specific surface area increase may be typically observed when the nanoparticles’ size decreases [73]. Increasing the Zr^4+^ content in the material up to 3.7% caused V_p_’s increase, and then drop when Zr^4+^ content reaches 5.6%, while S_p_ grew from 3.8 to 7.5 nm. The above observations may be concluded that prepared materials have mesoporous structures created by aggregates built of spherical TiO_2_ nanoparticles [74]. Undoubtedly, the structure of used precursors (isopropoxide and n-propoxide alkoxy chains) can act as semi-templates and induce pores’ growth in investigated materials [63].

#### 3.2.2. Physicochemical Analysis

**Figure 9:**
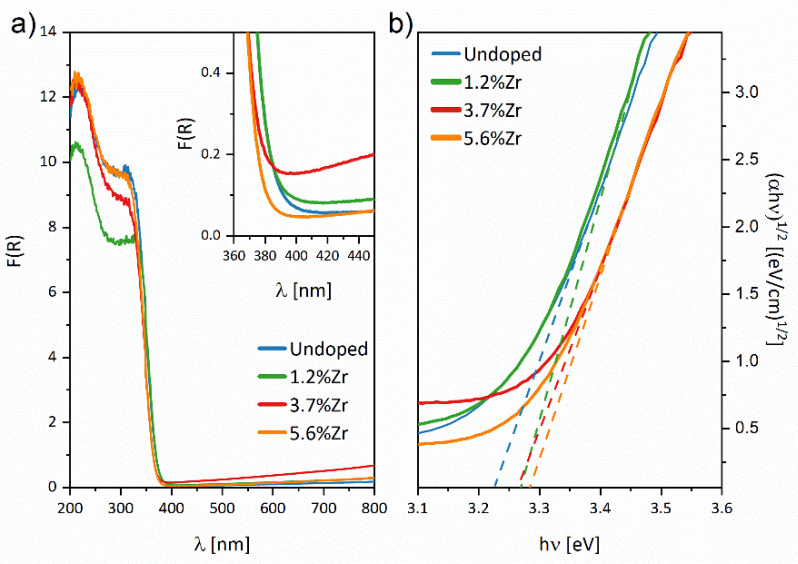
DRS spectra (**a**) calculated using the Kubelka–Munk function F(R), and indirect bandgap evaluation (**b**) for investigated samples.

Figure 9 shows the diffuse reflectance spectra (a), and indirect bandgap (b) plotted via *(αhυ)^1/2^* vs. *(hυ)* of nanoparticles deposited on FTO substrates. As shown in the DRS graphs, presented materials absorb light in the UV region (<400 nm) mainly. Nevertheless, after embedding Zr^4+^ ions, a blue shift was observed in the case of 3.7%Zr_FTO and 5.6%Zr_FTO samples and a significant absorption increase in the Vis light range with a maximum effect for 3.7%Zr_FTO sample. That is in good agreement with our theoretical calculations because, as shown in Figure 2d–i, introducing substituting Zr^4+^ ions or creating oxygen vacancies leads to increased energy bandgap (see Table 3). As mentioned above, the energy gap area’s additional energy level due to very low DOS does not affect the optical spectrum.

The calculated values of indirect bandgaps for TiO_2_ and Zr^4+^ ions doped materials with increasing Zr^4+^ ions content from 0 to 5.6% are 3.22, 3.27, 3.26, and 3.28 eV, respectively (see Table 6). It is worth paying attention to the fact that the growth of the bandgap value is nonlinear, which may be a result of several contradictory factors, including decreases in nanoparticle sizes (especially in nanofilms) [75], doping of Zr^4+^ ions [76], or the formation of defects [77]. Gnatyuk et al. also observed a similar disproportion and explained it as a molecular scale mixing of Zr^4+^ ions and ZrO_2_ species in the TiO_2_ [76]. It should be emphasized that computational calculation results are very close to the experimental ones. The most likely structures which should be taken into consideration are TiO_2_v(O) for pristine TiO_2_ (2 × 2 × 1 TiO_2_ v(O)) and substitutional Zr with oxygen vacancies present far from the doped ion (2 × 2 × 1 TiO_2_ Zr v(O) far) due to the combination of total atom energy and bandgap values. It should be noticed that quantum chemical calculations were performed for bulk materials. The experimental data were measured for nanoparticles with environment interaction moving the UV-vis spectra into the red spectral range. Therefore, since the bandgap width increased in the experimental data, the structure (2 × 2 × 1 TiO_2_ Zr v(O) far) with oxygen vacancies located far away from Zr^4+^ ions is most probable for presented materials.

**Figure 10:**
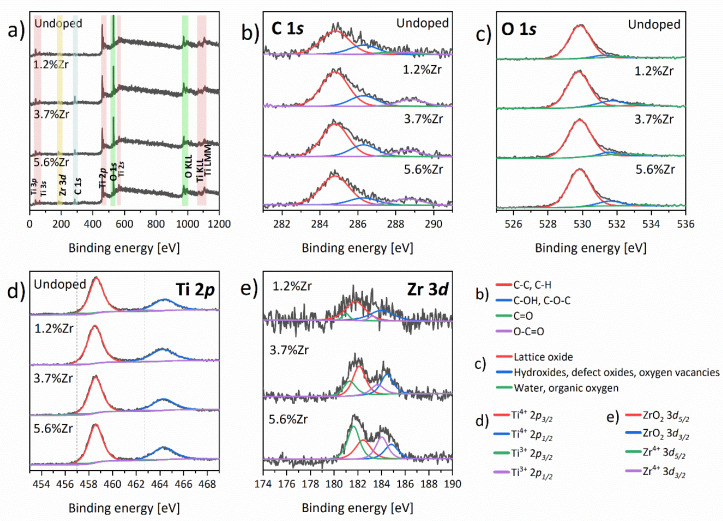
XPS spectra of nanoparticles annealed at 450 °C: (**a**) full scan, (**b**) C 1s, (**c**) O 1s, (**d**) Ti 2p (dotted line—Ti^3+^ 2p_3/2_ and 2p_1/2_), and (**e**) Zr 3d.

The X-ray photoelectron spectroscopy was performed to investigate the interaction between ions in as-prepared nanoparticles. Obtained XPS spectra are presented in Figure 10 and were analyzed and fitted using CasaXPS software (Casa Software Ltd, Teignmouth, UK). Three main bands discerned in the C 1s region (Figure 10b) may be resolved as alkyl, alcohol, and esters functional groups at 284.8, 286.3, and 288.8 eV, respectively. A minute amount of carbonyl group may also be observed at about 287.8 eV, except for the sample 5.6%Zr. The XPS spectra resolution in the C 1s region corresponds with our findings from the FTIR experiments described above. Furthermore, the intensity of C 1s signals increased in the doped materials compared with the undoped ones. The use of zirconium n-propoxide as a precursor of Zr^4+^ ions in the synthetic procedures may explain the higher concentration of organic residues in these samples. In the O 1s region (Figure 10c), two main bands may be identified. The main peak at 529.8 was assigned to the O^2-^ ions in the anatase TiO_2_ lattice, while 531.4 eV is typically ascribed as connected with hydroxyl groups, carbon impurities, or defective TiO_x_ [78]. A sample with a 1.2% concentration of Zr^4+^ ions also contained low water content. Figure 10d shows two characteristic 2p spin-orbit doublets of Ti^4+^ and Ti^3+^ (marked with dotted line). The two main peaks centered at 458.6 and 464.3 eV with a Ti 2p_1/2_ − Ti 2p_3/2_ splitting equally to 5.70 eV corresponds with Ti^4+^ ions in the anatase phase [79]. During the heating process, oxygen molecules are detaching from TiO_2_, leaving oxygen vacancies, and hence the resulting surplus of electrons reduces the Ti^4+^ ions to Ti^3+^ [80]. The reduced Ti^3+^ ions are detected at 457.0 and 462.7 eV with the same splitting value, which is also in good agreement with the literature [79]. It is worth taking into account the ratio of Ti^4+^/Ti^3+^peaks area. The significant difference occurred in the undoped TiO_2_ (1:0.079), then the embedding of Zr^4+^ ions led to a decrease in the number of defects on the titania nanoparticles’ surface. However, when Zr^4+^ content increases in the nanoparticles, more Ti^3+^ species are indicated: 1:0.016, 1:0.055, and 1:0.046, respectively. It may be elucidated by the discrepancies in the ionic radius of Ti^4+^ and Zr^4+^, which may cause some lattice distortion. Two Zr 3d_5/2_ peaks at about 181.8–182.4 eV and 181–181.6 eV may be observed in Figure 10e and it can be resolved as the Zr^4+^ ions of the ZrO_2_ lattice and as a Zr^4+^ in Ti_1−x_Zr_x_O_2_ crystals, respectively [81]. The 3d_3/2_ signals were detected at 184–184.8 and 183.2–184 eV again for Zr^4+^ ions and ZrO_2_ in TiO_2_ matrices, correspondingly. It should be noted that with a higher content of Zr^4+^ ions in the samples, the shift occurred for all signals. Yu et al. calculated these two signals’ band ratio area to establish Zr^4+^ amount in Ti_1−x_Zr_x_O_2_ materials. Based on this paper, we determined that 0.127, 0.509, and 1.337% of Zr^4+^ ions doped the TiO_2_ structure. The detailed data of the percentage distribution of particular peaks in the XPS spectra registered for the materials presented are collected in Table S4.

The valence band positions were determined for two representative materials: bare TiO_2_ and doped with 3.7% Zr^4+^ ions based on the UPS spectra. The work functions of nanomaterials surfaces were calculated using the equation:

(9) $$\phi_{S}=h\upsilon -(E_{cut-off}-E_{Fermi}).$$

**Figure 11:**
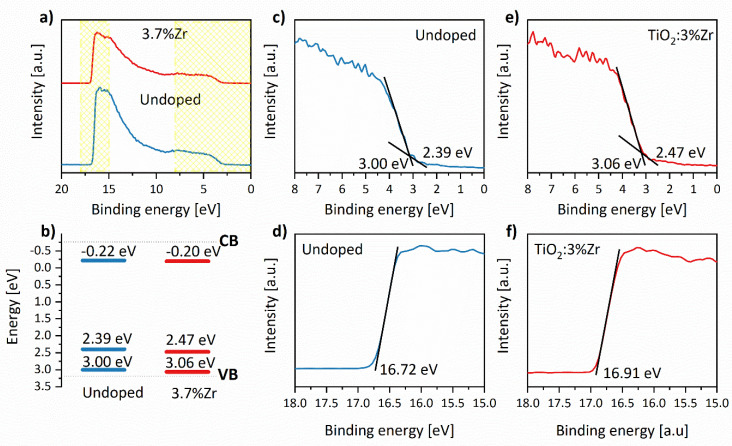
UPS spectra of undoped TiO_2_ (**a**,**c**,**d**) and nanomaterial with 3.7% content of Zr^4+^ ions (**a**,**e**,**f**) with a scheme of the energy structure band (**b**). The enlargements of valence bands (**c**,**e**) and secondary electron cut-offs (**d**,**f**) are shown.

The first term is related to photons’ He I energy (21.2 eV) applied in the UPS measurements, and the second one is the secondary electron cut-off energy. Therefore, based on the above results after doping with Zr^4+^ species, work-function decreases from 4.28 to 4.09 eV, respectively. The value of 4.28 eV for undoped nanomaterial is in good agreement with the literature [82,83], whereas 4.09 eV points out introducing dopant ions caused by minimizing charge injection barriers [82]. Factors that affect the work’s function are, among others, doping or contamination on crystallites surfaces [84]. Therefore, that can explain the difference between these two materials. Figure 11b presents a scheme of the energy band structure prepared by combining the UPS (Figure 11c,e) and DRS (Table 6) results. As can be seen, there are slight differences, about 0.02 eV in the conduction band and 0.06 eV in the valence band values. The above scheme’s band shape is in excellent agreement with the theoretical calculation shown in Figure 3. In addition, extra bands were detected in both UPS spectra (Figure 11c,e) at 2.39 eV for undoped TiO_2_ and 2.47 eV for TiO_2_:3.7%Zr. The experimental results again coincide with computational calculations (Figure 2e,h,i), and recorded bands stem from oxygen vacancies present in both nanomaterials.

**Figure 12:**
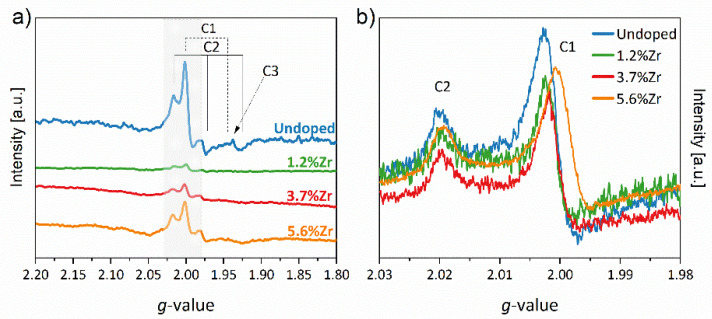
X-band EPR spectra (**a**) with an enlargement (**b**) recorded at 77 K with distinct three components.

The paramagnetic phenomenon of the nanomaterials annealed at 450 °C was tested by Electron Paramagnetic Resonance (EPR). The EPR spectra depicted in Figure 12a,b show three distinguishable paramagnetic centers (C1-3). It is a well-known fact that with the increment of the temperature, the number of vacancies also increases to the moment of phase transformation by thermal depletion of oxygen [80]. This statement may justify the appearance of defects in the matrices of TiO_2_ calcinated at 450 °C. The differences in shifts and intensity observed in the EPR spectra (Figure 12b) originate from the measurements’ low resolution. A detailed study of the EPR spectra is beyond this paper’s scope, but still, component C1 line shape can indicate the Ti^3+^ ions embedded in a regular site of anatase lattice. Mohajernia et al. noticed a similar defect in TiO_2_ annealed at 700 °C in the air and described it as a Ti^3+^ with moderate tetragonal distortion g-tensor values equal g_x_ = 1.994, g_y_ = 1.994, and g_z_ = 1.944, which also corresponds to our findings [85]. The last signal of g_z_ tensor is not visible in the above graph, which may be related to a superimposition of highly disordered component C3 of Ti^3+^ species in the surface sites [86]. It is worth noticing that oxygen vacancies are also detected as anisotropic g-tensors of g_1_ = 2.017, g_2_ = 1.974, and g_3_ = 1.927. Under the above considerations, the situation that occurred in as-prepared samples may be described by the following equation:

(10) $$O^{2-}+2Ti^{4+}\to \frac{1}{2}O_{2}+v\left(O\right)+2Ti^{4+}+2e^{-}\to \frac{1}{2}O_{2}+v\left(O\right)+2Ti^{3+}$$

After thermal depletion of oxygen molecules, an excess of electrons stabilizes the resultant Ti^3+^ ions, consistent with XPS analysis. Furthermore, it can be seen that introduction of Zr^4+^ ions at the beginning caused the reduction of paramagnetic signals’ intensity and again increment with increasing of Zr^4+^ content. This observation correlates with XPS and XRF results. After metal ions doping, the rutile phase disappeared, which could be explained by lattice distortion induced by both v(O) and the presence of additional crystal structure. Notwithstanding, a higher dopant ions content again disturbed the crystal lattice, which can be explained by the differences in the ionic radius of Zr^4+^ and Ti^4+/3+^. The detailed data of g parameters observed in the EPR spectra of presented materials are collected in Table S5.

#### 3.2.3. Photovoltaic Characterization

**Figure 13:**
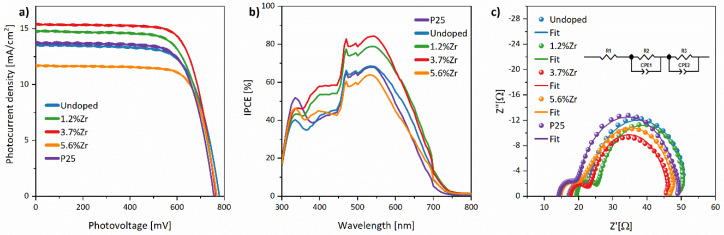
J-V characteristics (**a**), IPCE spectra (**b**), and Nyquist plots with inserted equivalent circuit scheme used to fit the EIS spectra (**c**) of investigated DSSCs.

The performance of DSSCs was investigated under AM 1.5G simulated sunlight by J-V characteristics and electrochemical impedance spectroscopy measurements. A commercial P25 material was tested as a reference. As shown in Figure 13a, the open-circuit photovoltage (V_OC_) value drop from 779.1 to 768.1 mV for undoped material and 3.7% Zr^4+^-doped TiO_2_, respectively. Notably, V_OC_ is a difference between the semiconductors’ conduction band Fermi level and the redox couple Nernst potential in the electrolyte [87]. As shown in Figure 2d,e,h,i, replacing Ti^4+^ ions with Zr^4+^ leads to the lower values’ conduction band’s shift. As the type of electrolyte remained unchanged during the whole experiment, the conduction band’s shift seems to be the most probable reason for the V_OC_ changes. Therefore, computational calculations again supported the experimental results and explained the reason for the V_OC_ drop. Moreover, the above result corresponds with the energy scheme (Figure 11b), combining results extracted from DRS and UPS analyses.

Another DSSC device parameter, short-circuit photocurrent density (J_SC_), mainly corresponding to the charge recombination/electron transports and the working electrode’s specific surface area, was also determined. As shown in Table 7, the more considerable Zr^4+^ ion content, the higher J_SC_ value, up to 15.47 mA/cm^2^ for 3.7% Zr^4+^ ions, and then dropped when Zr^4+^ concentration reaching 5.6%. Undoubtedly, the surface area increment in the investigated sample series corresponds to the dye loading (see N_dye_ value in Table 7) and caused enhancement of short-circuit photocurrent density. However, the J_SC_ sharp decline in the case of 5.6% Zr^4+^ may be caused by carbon residues, the presence of which has been confirmed by XPS and FTIR results described above. According to the literature data, carbon impurities significantly impact charge transport and recombination processes, leading to photocurrent density weakness [63].

It should be noted that the fill factor (FF) values registered for all devices are very high (>70%), which indicates that the semiconductor manufacturing process developed by our research group ensures high-quality cells. Fill factor is a crucial DSSC parameter and represents, among others, the energy loss caused by series resistance, electrolyte thickness (spacer influence), and counter electrode production quality (Pt layer) [88,89,90]. It may be observed that indeed the TCO resistance (R_1_) correlates with FF since the highest FF value was observed for DSSC with the lowest R_1_ values (see Table 7). In contrast, it is challenging to define the direct influence counter electrodes resistance (R_2_) due to the Nyquist plot fitting uncertainty.

The overall photon-to-current conversion efficiency (η) is the most measurable cell efficiency indicator based on other parameters. As shown in Table 7, similar DSSC performance, equal to 7.49 and 7.41%, have been registered for P25 nanopowder and bare TiO_2_ cells, correspondingly. Again, a gradual increase of η is observed up to 3.7% Zr^4+^ device, then drops to the lowest value of 6.74% for 5.6% Zr^4+^ device. The above effect may be a combination of several factors mentioned above, e.g., bandgap value, specific surface area, and dye loading. On the one hand, the incorporation of Zr^4+^ ions causes imperfection in the crystal lattice, improving the cells’ photoelectrochemical activity; on the other hand, defect surplus may promote charge recombination and decrease the DSSC efficiency [21]. To sum up, the increasing photon-to-current conversion efficiency supports the appropriateness of introducing Zr^4+^ dopants into TiO_2_ (up to 3.7% content of Zr^4+^ ions).

The incident photon-to-current efficiency (IPCE) shown in Figure 13b reveals that the typical N3 dye profile corresponds to its light absorption spectrum with a maximum at 540 nm characteristic for metal-to-ligand charge transfer bands [91]. The obtained IPCE values are in good agreement with the overall efficiency of DSSC.

Electrochemical impedance spectroscopy (EIS) was also conducted to understand the electron transport mechanism in DSSC better. Generally, Nyquist plots consist of four elements. The first one is a series resistance linked to the FTO substrate (R_1_). In contrast, the small semicircle corresponds to the high-frequency area is a combination of charge transfer resistance on the counter electrode (R_2_ = R_Pt_) and Helmholtz capacity (C_Pt_). The second bigger semicircle in the medium frequency range is related to recombination process resistance (R_3_ = Rct) and chemical capacity (C_µ_) at the TiO_2_ layer/dye/electrolyte interface. The last semicircle, called the Warburg element, at the low-frequency region, is the electrolyte impedance image (Z_d_), and it is not observed in Figure 13c. The EIS data collected in Table 7 were calculated based on the Nyquist plot fitting using the equivalent circuit scheme inserted in Figure 13c. This diagram is composed of resistors (R_1_—FTO and external circuit resistance, R_2_—Pt resistance, R_3_—TiO_2_ layer/dye/electrolyte recombination processes resistance), and constant phase elements (CPE1 and CPE2).

As described above, R_1_ values are related to the FTO and external circuit resistance, and they correlate with FF values extracted from J-V curves, while R_2_ values connected to the counter electrode are consistent one to another. The minor discrepancies in these two parameter values may be associated with the typical imperfect “hand-made” method of preparing DSSC in the presented research [32]. The more important in the EIS results is the R_3_ parameter assigned to the electron transport at TiO_2_/dye/electrolyte interface corresponding with the overall photoconversion efficiency. Indeed, this study has a noticeable tendency–the higher the R_3_ parameter, the lower η value. The one exception is observed in the case of 5.6% content of Zr^4+^ ions. Initially, the doping of Zr^4+^ ions leads to improving the electron transfer through the cell what can be seen in diminishing the R_3_ values, but then dropped concerning to the 5.6%Zr. Ünlü et al. noticed a similar situation in the Mn^2+^-doped TiO_2_ and described it as a structural problem, not only resulting from the recombination processes [92]. Based on this statement and our results, the increasing specific surface area with increasing zirconium content should be considered. Despite that, the highest amount of adsorbed dye molecules is connected with the standard P25 (which may be elucidated as the densest packed thin film in investigated series of DSSC), while the second one was 5.6%Zr cell with the value of 43.85 nmol/cm^2^. The mesoporous structure of TiO_2_:5.6%Zr^4+^ with 132.9 m^2^/g specific surface area and 7.5 nm pores size provides a larger surface for dye adsorption, leading to the aggregation of dye and photoconversion efficiency decline. It is worth noticing that amount of adsorbed dye is a decisive factor up to the particular level, after which the photoconversion efficiency reaches a *plateau* [93]. Another reason may be related to crystallites’ size because nanomaterial with a 5.6% content of Zr^4+^ ions indicates larger crystallites than 3.7% Zr^4+^ material. Moreover, the increment of Zr^4+^ and ZrO_2_ species on the TiO_2_ surface can also act as recombination centers even though a certain level of doping provides better contact TiO_2_ with dye molecules [94]. These considerations may be supported by the electron lifetimes calculated from the Bode plot. It can be shown that after doping the 1.2% of Zr^4+^ ions injected electron lifetime increase and then decline: 10.18, 15.96, 12.76, and 10.18 ms for TiO_2_:xZr^4+^ where x = 0, 1.2, 3.7, 5.6%, respectively. As shown in Table 7, the longer the electron lifetime, the higher photoelectric conversion efficiency with a slight difference between 1.2% and 3.7% content of Zr^4+^ ions in examined nanomaterials. It can be explained as a strong impact of injected electron lifetime on recombination and photon-to-current conversion processes [63]. Electron injection and recombination processes are contradictory mechanisms. Therefore, it is worth highlighting that DSSC can be affected by many factors, concluding that it is difficult to point out the exact reason for these small discrepancies.

## 4. Conclusions

In conclusion, inserting the Zr^4+^ ions into TiO_2_ lattice in substituted positions was obtained in this work. Nanopowders and working electrode calcination at 450 °C also led to the formation of defects in the titanium dioxide structure. Doping of Zr^4+^ ions or sintering titania film on FTO substrate caused crystal growth inhibition (below 10 nm in size). It also preserved emerging the rutile phase (observed in the undoped sample). These observations were in line with theoretical calculations that oxygen vacancies and Zr^4+^ ions doping caused stabilization of TiO_2_ structure without substantial cell parameter changes. Moreover, we showed that with increasing dopant ions content, the specific surface area increases and by extension, the amount of adsorbed dye on the TiO_2_ surface also increases. Furthermore, defects in the TiO_2_ crystal lattice induced the broadening of the energy bandgap and shifted the conduction band to lower energy. Again, experimental results were in excellent agreement with computational calculations. All of the factors mentioned above had an impact on the working principle of DSSCs. Shifting of conduction band caused decreasing of open-circuit photovoltage, which in case of 3.7% of Zr^4+^ ions content occurred to be optimized for effective electron transfer injection. It is worth noticing that as a result, short-circuit photocurrent density was enhanced to the 15.47 mA/cm^2^, and recombination processes on the TiO_2_/dye/electrolyte interface were suppressed. Finally, we reported for the first time, highly efficient DSSC built with photoanode consists of substituted Zr^4+^ species in TiO_2_.

## Figures and Tables

**Table 1:** List of photovoltaic parameters of DSSCs based on a photoanode prepared using titanium oxide doped with Zr^4+^ ions.

Dopant	Phase	Annealling Temp. [°C]	JSC [mA/cm2]	VOC [V]	FF [%]	η [%]	Ref.
5% Zr	Anatase	450	0.017	0.71	20	<1	[19]
10% Zr	Anatase	400	0.13	0.45	0.34	0.02	[20]
5% Zr	Anatase	600	10.66	0.64	61	4.16	[24]
0.5% Zr	Anatase	450	21.83	0.65	53	7.52	[21]
2% Zr	Anatase	500	6.28	0.787	63.2	3.12	[22]
0.3% Zr	Anatase	500	12.91	0.7239	72	6.7	[23]
1% Zr	Anatase	500	7.74	0.82	71.1	4.51	[25]
3.7% Zr	Anatase + Brookite	450	15.47	0.7641	72.97	8.63	This study

**Table 2:** Parameters of the unit cell of the a-TiO_2_ and t-ZrO_2_ stoichiometric and defected crystals obtained relaxing structure by applying DFT/PBE+U (U_Ti_ = U_Zr_ = 6 eV and J_Ti_ = J_Zr_ = 1 eV) method and compared to experimental data.

Structure	a (a = b) [Å]	c [Å]	c/a	Total Energy/Atom [eV]
a-TiO_2_	3.85	9.71	2.52	−8.74
	3.79 [38,39,40]	9.51	2.51	−
t-ZrO_2_	3.67	5.21	1.50	−9.27
	3.59 [36]	5.19	1.44	−
	3.64 [37]	5.27	1.45	−
2 × 2 × 1 TiO_2_	7.69	9.70	2.52	−8.73
2 × 2 × 1 TiO_2_ v(Ti)	7.72	9.62	2.49	−8.43
2 × 2 × 1 TiO_2_ v(O)	7.68	9.72	2.53	−8.70
2 × 2 × 1 TiO_2_Zr *	7.72	9.77	2.53	−8.78
2 × 2 × 1 TiO_2_ Zr v(O) *	a = 7.72 b =7.69	9.78	2.49 2.51	−8.74
2 × 2 × 1 TiO_2_ Zr v(O) * far	a =7.72 b = 7.70	9.79	2.56 2.54	−8.75
2 × 2 × 1 TiO_2_+Zr **	a = 7.73 b = 7.79	9.70	2.51 2.49	−8.70
2 × 2 × 1 TiO_2_+Zr v(O)	a = 7.73 b = 7.84	9.72	2.51 2.48	−8.68
2 × 2 × 1 TiO_2_+Zr v(O) far	a = 7.69 b = 7.86	9.72	2.53 2.47	−8.67

* 2 × 2 × 1 TiO_2_Zr ≡ Ti_0.94_Zr_0.06_O_2_. ** 2 × 2 × 1 TiO_2_+Zr ≡ TiO_2_ + Zr.

**Table 3:** The energy of the bandgap calculated by using the DFT/PBE+U method for all modeled structures (the same description as in Table 2).

Structure	E_bg_ [eV]
a-TiO_2_	3.16
2 × 2 × 1 TiO_2_	3.21
2 × 2 × 1 TiO_2_ v(O)	3.29
2 × 2 × 1 TiO_2_ v(Ti)	3.19
2 × 2 × 1 TiO_2_ Zr	3.21
2 × 2 × 1 TiO_2_ Zr v(O)	3.26
2 × 2 × 1 TiO_2_ Zr v(O) far	3.36
2 × 2 × 1 TiO_2_ + Zr	3.06
2 × 2 × 1 TiO_2_ + Zr v(O)	3.16
2 × 2 × 1 TiO_2_ +Zr v(O) far	3.27

**Table 4:** Cell parameters of Zr^4+^ ions doped TiO_2_ nanopowders and layers on FTO.

Sample	D_hkl_ [nm]	a = b [Å]	c [Å]	Cell volume [Å]
TiO_2__FTO	7.61	3.7948	9.5616	134.5931
TiO_2_:1.3%Zr_FTO	6.73	3.7896	9.5204	133.5283
TiO_2_:3.7%Zr_FTO	5.91	3.7976	9.5368	134.6059
TiO_2_:5.6%Zr_FTO	6.75	3.7936	9.5520	134.5368
TiO_2_ (60 °C, 12 h)	4.89	3.8018	9.5060	137.5812
TiO_2_ (450 °C, 1 h)	6.44	3.7868	9.4664	135.9226
TiO_2_ (450 °C, 2 h)	12.85	3.7884	9.5096	136.5429
TiO_2_:1.3%Zr	8.33	3.7834	9.4808	135.7306
TiO_2_:3.7%Zr	6.56	3.7888	9.4976	136.4813
TiO_2_:5.6%Zr	6.62	3.7920	9.5176	136.7810

**Table 5:** Structural parameters of mesoporous TiO_2_ nanopowders annealed at 450 °C for 2 h.

Sample	A_BET_ [m^2^/g]	V_p_ [cm^3^/g]	S_p_ [nm]
Undoped	69.4	0.156	3.8
1.2%Zr	89.1	0.238	5.5
3.7%Zr	101.0	0.325	5.5
5.6%Zr	132.9	0.280	7.5

**Table 6:** Indirect energy bandgap values extracted from Tauc’s plots.

Sample	E_bg_ [eV]
Undoped	3.22
1.2%Zr	3.27
3.7%Zr	3.26
5.6%Zr	3.28

**Table 7:** Photoelectrochemical parameters of the DSSC based on synthesized nanoparticles.

Sample	V_OC_ [mV]	J_SC_ [mA/cm^2^]	FF [%]	η [%]	N_dye_ [nmol/cm^2^]	IPCE [%]	R_1_ [Ω]	R_2_ [Ω]	R_3_ [Ω]	τ [ms]
P25	756.1	13.62	72.71	7.49	46.55	68.1	14.29	4.985	30.24	5.49
Undoped	779.1	13.50	70.39	7.41	16.62	68.6	17.34	4.785	30.79	10.18
1.2%Zr	767.1	14.68	70.66	7.96	27.36	78.8	18.99	6.682	26.26	15.96
3.7%Zr	764.1	15.47	72.97	8.63	33.76	84.3	17.28	6.246	22.70	12.76
5.6%Zr	768.1	11.63	75.45	6.74	43.85	63.8	13.6	10.46	24.21	10.18

## Data Availability

Data are available on request at corresponding authors.

## Supplementary Materials

The following are available online at https://www.mdpi.com/article/10.3390/ma14112955/s1, Figure S1: Unit cell of a-TiO_2_ crystal structure (blue—Ti atoms, red—O) (a,b) and t-ZrO_2_ crystal structure (—Zr atoms, red—O) (c), Figure S2: XRF spectra of nanopowders calcined at 450 °C, Figure S3: The E_g_ enlargement of Raman spectra: nanopowders (a) and FTO nanoparticles (b), Figure S4: SEM-EDS analysis of TiO_2_:3.7%Zr, Table S1: The wavenumber assignment of particular modes observed in Raman spectra, Table S2: Detailed data concerned the Eg mode extracted from the Raman spectra, Table S3: Infrared data of samples dried at 60 °C and after calcination at 450 °C, Table S4: Summary of the percentage distribution of particular peaks in the XPS spectra, Table S5: Comparison of the g parameters observed in the spectra (*—not visible in the graph).
