# Supplementary material for: How Can the Introduction of Zr4+ Ions into TiO2 Nanomaterial Impact the DSSC Photoconversion Efficiency? A Comprehensive Theoretical and Experimental Consideration
Source: Materials (Basel). 2021 May 30;14(11):2955. doi: 10.3390/ma14112955 (PMC8198604; doi:10.3390/ma14112955)
Supplement: Supplementary file 1 [file materials-14-02955-s001.zip › materials-1218106-SM.pdf]

materials

MDPI

Supplementary Materials

How Can the Introduction of Zr^{4+} Ions into TiO_2 Nanomaterial Impact the DSSC Photoconversion Efficiency? A Comprehensive Theoretical and Experimental Consideration

Aleksandra Bartkowiak [1,2,*], Oleksandr Korolevych [3], Gian Luca Chiarello [2], Małgorzata Makowska-Janusik [3] and Maciej Zalas [1,*]

[1] Faculty of Chemistry, Adam Mickiewicz University, Poznań, Uniwersytetu Poznańskiego 8, 61-614 Poznań, Poland;
[2] Department of Chemistry, University of Milan, Via Golgi 19, 20133 Milano, Italy; gianluca.chiarello@unimi.it
[3] Faculty of Science and Technology, Jan Długosz University, Al. Armii Krajowej 13/15, 42-200 Częstochowa, Poland; oleksandr.korolevych@ujd.edu.pl (O.K.); m.makowska@ujd.edu.pl (M.M.-J.)
* Correspondence: aleksandra.bartkowiak@amu.edu.pl (A.B.); maciej.zalas@amu.edu.pl (M.Z.)

Table of contents

Theoretical calculation – structural properties 2
XRF 2
Raman 3
SEM 4
XPS 6
EPR 6

Theoretical calculation – structural properties

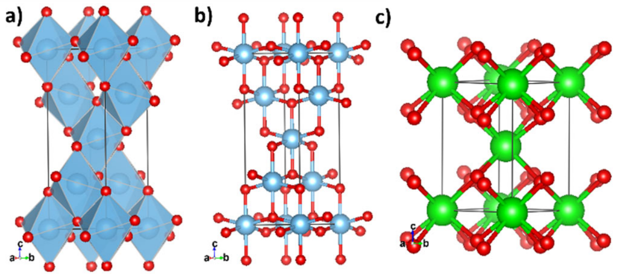

Figure S1. Unit cell of a-TiO_2 crystal structure (blue—Ti atoms, red—O) (**a**,**b**) and t-ZrO_2 crystal structure (green—Zr atoms, red—O) (**c**).

XRF

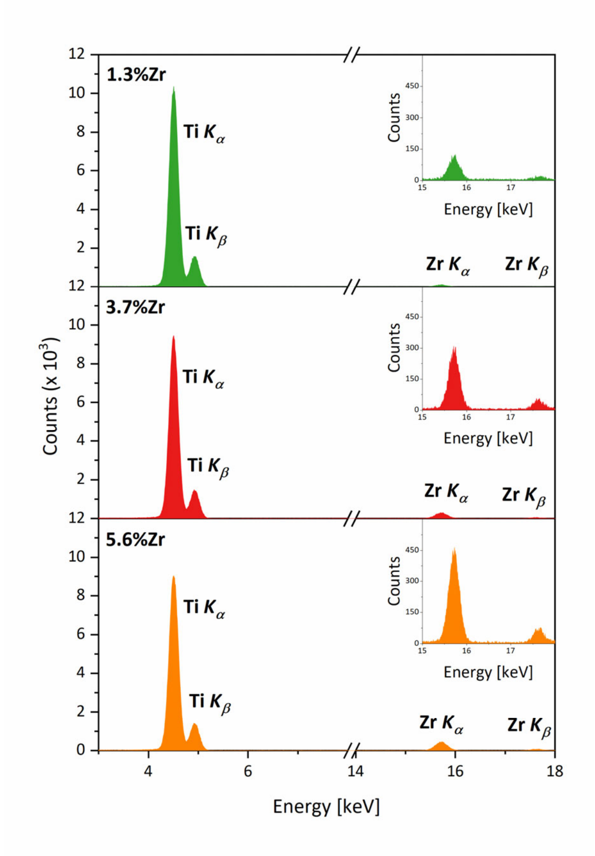

Figure S2. XRF spectra of nanopowders calcined at 450°C.

Raman

Table S1. The wavenumber assignment of particular modes observed in Raman spectra.

Sample		$E_{g(1)}$ [cm⁻¹]	$E_{g(2)}$ [cm⁻¹]	A_{1g} [cm⁻¹]	B_{1g} [cm⁻¹]	B_{2g} [cm⁻¹]	$B_{1g(1)}$ [cm⁻¹]	$A_{1g}/B_{1g(2)}$ [cm⁻¹]	$E_{g(3)}$ [cm⁻¹]
Undoped	FTO	144.7	196.7	245.5	322.7	367.7	398.1	519.5	639.6
1%Zr	FTO	146.3	194.3	243.9	323.8	366.6	398.1	520.9	639.4
3%Zr	FTO	146.8	-	247.2	324.9	369.7	397.2	521.6	639.1
5%Zr	FTO	146.4	191.8	246.3	324.1	367.6	396.2	521.4	638.9
Undoped	Powder	143.5	195.2	244.7	323.1	368.7	396.7	518.4	638.3
1%Zr	Powder	145.8	194.6	246.3	323.6	367.0	397.4	521.1	639.3
3%Zr	Powder	146.7	192.6	246.5	322.9	369.7	396.6	521.9	639.0
5%Zr	Powder	146.4	-	248.3	321.0	366.3	395.5	521.9	639.1

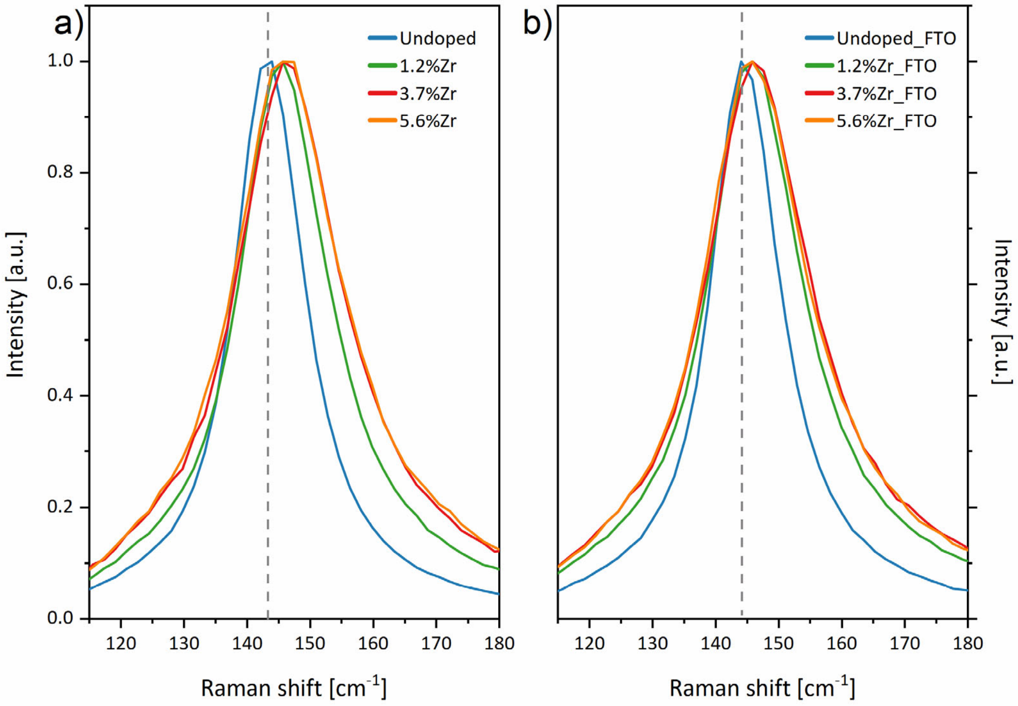

Figure S3. The E_g enlargement of Raman spectra: nanopowders (**a**) and FTO nanoparticles (**b**).

Table S2. Detailed data concerned the Eg mode extracted from the Raman spectra.

Sample		$E_{g(1)}$ [cm^{-1}]	FWHM	Phonon lifetime τ [ps]
Undoped	FTO	144.7	13.6	0.390
1%Zr		146.3	18.1	0.293
3%Zr		146.8	20.1	0.264
5%Zr		146.4	20.2	0.263
Undoped	Powder	143.5	13.6	0.390
1%Zr		145.8	17.4	0.305
3%Zr		146.7	20.1	0.264
5%Zr		146.4	20.4	0.260

SEM

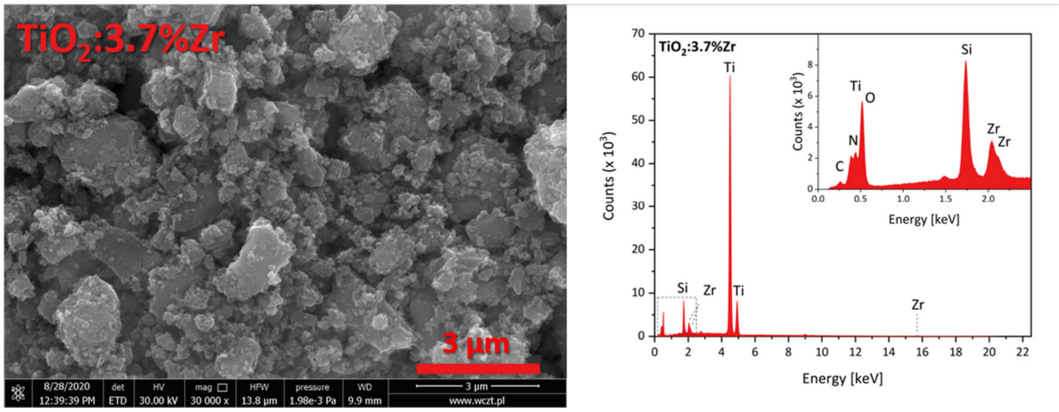

Figure S4. SEM-EDS analysis of TiO_2:3.7%Zr.

FTIR

Table S3. Infrared data of samples dried at 60°C and after calcination at 450°C.

Assignment	Samples Dried at RT				Samples Calcinated at 450°C			
	Undoped	1.2%Zr	3.7%Zr	5.6%Zr	Undoped	1.2%Zr	3.7%Zr	5.6%Zr
Stretching vibration νM-O	480–625	485–625	475–625	475–625	475–625	470–625	470–630	465–630
Stretching vibration νM(O-C)	1160	1160	1160	1155	-	-	-	-
Asymmetrical stretching vibration ν_{asym}C-O (esters)	-	-	1228-1233	1213-1234	-	-	-	-
Bending (deformation) vibration δCH	1384	1384	1384	1384	1383	1383	1384	1384
Symmetrical stretching vibration ν_{sym}COO	1430	1440	1446	1446	-	-	-	-
Asymmetrical stretching vibration ν_{asym}COO	1540	1539	1539	1540	-	-	-	-
Bending vibration δOH	1627	1627	1628	1628	1628	1624	1628	1624
Stretching vibration νC=O	1766	1768	1768	1769	-	-	-	-
CO_2 atmospheric	2426	2426	2426	2426	-	-	-	-
Asymmetrical stretching vibration $\nu_{asym}CH_2$	2854	2853	2854	2850	2850	2852	2852	2852
Symmetrical stretching vibration $\nu_{sym}CH_2$	2924	2926	2926	2925	2924	2923	2922	2923
Symmetrical stretching vibration $\nu_{sym}CH_3$	2973	2970	2972	2974	2960	2964	2960	2966
Stretching vibration νOH	3200–3370	3200–3360	3200–3364	3200–3345	3200–3300	3200–3300	3200–3300	3200–3300

XPS

Table S4. Summary of the percentage distribution of particular peaks in the XPS spectra. .

Peak	Band	Undoped	1.2%Zr	3.7%Zr	5.6%Zr
		Concentration [%]	Concentration [%]	Concentration [%]	Concentration [%]
C 1*s*	C-H/C-C	62.91	65.97	63.88	68.12
	C-OH/C-O-C	24.97	20.15	22.87	16.88
	C=O	6.62	0.71	0.72	0
	O-C=O	5.50	13.16	12.53	15.00
O 1*s*	O^{2-}	89.51	84.01	86.49	85.64
	OH^-	10.49	11.35	13.51	14.36
	Water	-	4.64	-	-
Zr 3*d*	Zr $3d_{5/2}$	-	6.76	20.23	34.34
	Zr $3d_{5/2}$		4.51	13.49	22.89
ZrO_2 3*d*	Zr $3d_{5/2}$		53.24	39.77	25.67
	Zr $3d_{5/2}$		35.49	26.51	17.11
Ti 2*p*	Ti^{4+} $2p_{3/2}$	61.66	65.55	63.09	63.67
	Ti^{4+} $2p_{1/2}$	30.95	32.90	31.67	31.96
	Ti^{3+} $2p_{3/2}$	4.92	1.04	3.49	2.91
	Ti^{3+} $2p_{1/2}$	2.47	0.52	1.75	1.46

EPR

Table S5. Comparison of the g parameters observed in the spectra (*—not visible in the graph).

Sample	Center	g_1	g_2	g_3
Undoped	C2	2.017	1.974	1.927
	C1	1.995	1.995	*
1.2%Zr	C2	2.017	1.973	1.921
	C1	1.994	1.994	*
3.7%Zr	C2	2.018	1.976	1.921
	C1	1.994	1.994	*
5.6%Zr	C1	2.018	1.973	1.925
	C2	1.995	1.995	*
